# Sources of exhaustion among hospital conventional radiology technicians in Santa Catarina, Brazil

**DOI:** 10.5327/Z1679443520190386

**Published:** 2019-12-01

**Authors:** Tiago Jorge Anderson, Tatiana Martins, Francine Lima Gelbcke

**Affiliations:** 1 Department of Nursing, Polydoro Ernani de Santiago University Hospital - Florianópolis (SC), Brazil. Department of Nursing Polydoro Ernani de Santiago University Hospital Brazil; 2 Graduate Nursing Program, Universidade Federal de Santa Catarina - Florianópolis (SC), Brazil. Universidade Federal de Santa Catarina Graduate Nursing Program Universidade Federal de Santa Catarina Brazil

**Keywords:** radiology, occupational health, radiology department, hospital, occupational diseases

## Abstract

**Background:**

Health care workers, including radiology technicians, are exposed to work burdens liable to cause exhaustion.

**Objective:**

To describe sources of exhaustion within the work process of technicians at a hospital department of conventional radiology in Santa Catarina, Brazil.

**Methods:**

Qualitative, descriptive and exploratory study in which data were collected from 12 radiology technicians by means of semi-structured interviews. The data were subjected to thematic analysis.

**Results:**

Three sources of exhaustion stood out: musculoskeletal, represented by shoulder and back pain, mental, corresponding to stress and emotional and psychological overload, and respiratory, manifested as colds, pneumonia and rhinitis.

**Conclusion:**

The present study makes visible sources of exhaustion within the work process of radiology technicians, which are similar to those that affect other categories of health care workers.

## RESUMO |

**Introdução:**

Os profissionais da saúde estão expostos a cargas de trabalho que interagem com o corpo e podem gerar desgastes. Entre esses profissionais, temos os das técnicas radiológicas.

**Objetivo:**

Descrever os desgastes presentes no processo de trabalho dos profissionais das técnicas radiológicas em serviço hospitalar de radiologia convencional no estado de Santa Catarina.

**Metodologia:**

Trata-se de uma pesquisa qualitativa, descritiva e exploratória. A técnica de coleta de dados foi entrevista semiestruturada, realizada com 12 profissionais técnicos em radiologia. A análise ocorreu por meio da análise temática

**Resultados:**

Destacaram-se três categorias de desgaste: os osteoarticulares, que foram manifestados por dores no ombro e na coluna vertebral; os psíquicos, que abordaram o estresse e o desgaste emocional e psicológico; e os do sistema respiratório, manifestando-se por meio de resfriado, pneumonia e rinite.

**Conclusões:**

A pesquisa permitiu dar visibilidade aos desgastes ocasionados pelo processo de trabalho dos profissionais das técnicas radiológicas em radiologia convencional. Evidenciou-se que esses desgastes são similares aos de outras categorias profissionais na área da saúde.

## INTRODUCTION

According to Marx, work reflects changes human beings make in nature to meet their own needs. Indeed, the biological evolution of humankind can be seen as an adaptation of the human body to work, in as much as the modes of production and appropriation of tools and the products of work evolved variably in different times and places^1^. Work has an ontological dimension expressed as the historical ways human beings organized themselves within society. This fact is clear when one looks at past history, particularly at the shift from the food-collecting to the technological and industrial society.

Work also has substantial influence on individual and social illness. As observed by Mendes and Waismann^2^ “the transformations which led to work-related diseases are embodied in the production processes proper to each historical era and their associated sociotechnical possibilities.”

This relationship between work and illness is long known. In antiquity, Plato described exhaustion among professional athletes and Aristotle among professional horsemen. Plautus noticed posture problems among artists and tailors, leading to serious sequelae in some cases. In the 15th century, Ellenbog described cases of exhaustion among miners and goldsmiths, and in the 1500s, Agricola described miner’s asthma. The following century, Bernardino Ramazzini published *Diseases of Workers*, in which he established a relationship between work and disease among individuals or social groups^2^.

Laurell and Noriega’s work on Latin American social medicine is a reference for studies on the relationship between work and health/disease. These authors conceive of the health-disease phenomenon as a dialectical process in intimate correlation with the social context, more particularly with the effects of changes in social circumstances over time. Approaching work from a Marxist perspective, they define it as the core category in the analysis of the health-disease process in which notions such as work burden and exhaustion are used to identify the disease patterns exhibited by different groups. In this regard, work burdens are understood as components of the work process which dynamically interact among themselves and also with the physical body of workers. Exhaustion, in turn, is defined as “loss of effective and/or potential biological and mental ability.”^3^

The patterns of exhaustion proper to different occupational groups derive from the particular characteristics of the corresponding work process^3^. The relevance of research in occupational health precisely derives from a more thorough understanding of how work contributes to illness among different occupational groups, given that work has a determinant role in the health-disease process^4^.

The main characteristics of the job of health care workers depend on the fact they deal with human lives and that the product of work is consumed simultaneously to its very production, which distinguishes this from other production activities. Health care workers, therefore, should be healthy to ensure high-quality care and prevent iatrogenesis^5^.

Health care workers comprise several occupational groups, including radiology technicians and technologists. A study performed by Anderson^6^ found that beyond the common burdens associated to hospital work, this group is also exposed to factors inherent to the work process in radiology. Such risks include: exposure to ionizing radiation, large numbers of patients, working with broken equipment, exposure to body fluids, ergonomic risks related to handling weights, and accelerated pace of work^7^.

An integrative review^8^ located only seven articles on the health of workers in radiology departments, even though according to the National Council of Radiology Technicians this group comprises 116,016 workers in Brazil^9^.

As a function of the need to investigate the occurrence of exhaustion in this considerable population of workers and the few studies currently available, the aim of the present study was to describe sources of exhaustion among technicians in a department of conventional radiology at a hospital in Santa Catarina, Brazil.

## METHODS

The present qualitative, exploratory and descriptive study was conducted at a medium-to-high complexity general hospital, considered a referral center for orthopedics and traumatology, and administered by the Secretariat of Health of Santa Catarina^10^. We selected this particular hospital as a function of the high rate of radiological tests performed for orthopedic patients.

The study population comprised all the workers allocated to the departments of radiology and diagnostic imaging with jobs related to conventional radiology, to a total of 14. However, only 12 workers participated in semi-structured interviews, because two were on vacation/maternity leave and were excluded from the study.

Sociodemographic and occupational variables included sex, age, educational level, years in the job, work shift, having more than one job and overtime work. In the interviews we focused on burdens and risks inherent to the participant’s job. The interviews were performed in the workplace, taped, transcribed and presented to the participants for validation.

In data analysis we followed the thematic analysis method developed by Braun and Clarke^11^, which enables detecting, analyzing and describing themes, as well as interpreting several aspects related to the subject of interest^12^.

In compliance with the National Health Council Resolution no. 466/2012, the present study was approved by the research ethics committee of Universidade Federal de Santa Catarina, ruling no. 1,020,563. The participants signed an informed consent form. To protect their anonymity, the participants were attributed alphanumerical codes — I (interviewee) and a number indicating their order in the interviews.

## RESULTS

About 58.3% of the participants were female and 41.7% male; 8.3% were aged 20 to 30, 50% 30 to 40, 33.3% 40 to 50 and 8.4% 50 to 60. About 66.7% of the participants had completed secondary school and 33.3% had attended higher education. About 16.7% of the participants had worked 0 to 10 years in their current job, 75% 10 to 20 years and 8.3% 20 to 30 years. About 91.7% of the sample worked overtime, while 8.3% did not. About 33.3% of the participants had a second job, while 66.7% did not. Half of the sample (50%) worked in the morning and the other half in the afternoon.

Analysis of interviews pointed to three main sources of exhaustion: musculoskeletal, mental and respiratory. Other problems were less frequently mentioned and were clustered together as “other” for the purpose of analysis.

Musculoskeletal complaints included pain and injuries, most frequently involving the shoulders and spine, as a result of the need to handle excessive weights during the handling and transport of patients and equipment. These conditions were reported by 9 out of the 12 participants, which rate indicates this is a highly relevant occupational health problem among conventional radiology workers.

The participants’ narratives point to problems derived from exposure to physiological burdens, as is shown next:

And sometimes normal pain, pain in the shoulder, due to excess weight, because we don’t have some equipment, we don’t have a table that matches the stretchers used by firefighters, sometimes we have to climb on tables, there’s all this ergonomic work that causes pain in the shoulders, pain in the back. Moving heavy patients (I2).

I feel my shoulder, a burning in the shoulder, perhaps like inflammation, tendinitis, something like that. I associate this pain to changing cassettes when scans are going to be made, each image acquisition, or also to carrying and handling the tube, the test panels, they tend to lock, the equipment is always heavy because it hasn’t been duly calibrated, sometime the screws get loose, as the maintenance department says, and the rail is too heavy to handle. Besides the cassettes, depending on the number of tests, sometimes patients with multiple trauma, too many images to acquire, a lot of cassettes to carry, it gets very heavy. Even when few, like one or two spine tests, each cassette weights like about 2 kg, it’s a lot of weight (I5).

Sometimes you feel pain in the back, pain in the shoulder […] Tor lift heavy patients, in ICU three people do this: two lift him and you place it into position (I9).

Having to lift the patient, that which usually might cause injury to the shoulder or the back (I10).

Eight of the 12 participants reported also mental problems, including emotional and psychological stress which we categorized as mental exhaustion. The participants associated these disorders with having to deal with large numbers of patients, poor working conditions, little cooperation among staff members, having more than one job, having to work with sick people, and ethical issues related to patient exposure to ionizing radiation:

The cause of exhaustion is this: when there’re too many patients and the equipment has some problems, or anything like that, it causes a lot of emotional stress to me, because I get involved with the patient’s conditions, I want to help (I2).

Mental exhaustion, yes, we do feel, even because we work with sick, sensitive people, who have to wait long, are suffering, we end by…, I don’t know, as human beings we end by feeling sorry, even when we grow accustomed, but seeing that all too often, we feel it (I4).

The emotional side has to do with the number of patients, the work environment, sometimes, so to speak, too little collaboration among the staff, and the number of patients, who sometimes put pressure on us to do everything quickly, and there are too many of them for one single technician, they always blame us because it takes so long (I5).

I get stressed, I don’t have too much patience! But, what I get to see is that my colleagues who have also other jobs, they have in a more severe degree of stress, because they slept bad the night before, suffer other burdens, hindrances, tasks, and are already tired when they get here to work. Each one comes with their own stress, their own personal burdens, and they get here, and the infrastructure isn’t OKl, the demands are huge (I7).

Still in regard to mental exhaustion, it should be noticed that a large part of the participants worked overtime and/or had a second job.

Five out of the 12 participants reported respiratory complaints, including colds, pneumonia and rhinitis, which they associated to exposure to extreme temperatures, lack of periodic maintenance of air conditioners, work overload and a drop in immunity:

Colds once and again, which I believe have to do with the air conditioner, for lack of cleaning, or it’s too cold in one room and too hot in another room, and we keep going from one to the other and back all day long (I3).

Once I had pneumonia […] you think you got the flu, pain in the evening and in the weekend for about 48/60 hours, coughing all the time, I spent the full night coughing, three nights […] Then I asked my colleagues to take an x-ray, there was a spot in the lung, a very large one […] I showed it to the radiologist, he diagnosed pneumonia, I went to the occupational doctor, had to stay one week at home, then I did another x-ray test for control and was OK, I had taken antibiotics… I stayed one week at home (I9).

Rhinitis is missing! […] even because the room has no ventilation […] we’re exposed to air conditioning and get rhinitis (E2).

About 8.3% of the participants reported also other problems, including labyrinthitis in relation to the night-shift work, falls on wet floor, and scabies following contact with a patient.

## DISCUSSION

In the present study we evidence for the first time the main burdens associated with the work of radiology technicians. Previous studies on the health of this group focused on their occupational health as a whole, without particular attention to job specific aspects. According to Laurell and Noriega, identifying burdens and risks associated with definite production processes enables the implementation of prevention and health promotion measures targeting particular populations of workers^3^. Therefore, identifying the weaker points of definite occupational groups is indispensable to develop occupational health actions. Understanding the factors which affect radiology technicians enables public health actions targeting this group. According to the results of the present study, they are mainly represented by musculoskeletal, mental and respiratory problems.

Musculoskeletal disorders were the most frequently mentioned by the participants, particularly shoulder and back pain in association with handling weights and equipment. In the study by Coutinho^12^ with 67 radiology technicians, 58% of the sample observed that the tools used in their job posed risks to health and had influence on the occurrence of repetitive strain injury and musculoskeletal disorders. According to other authors, upon providing care to multiple trauma victims, radiology technicians have to handle gurneys and conventional radiology tables with fixed height. Problems are also posed by broken gurneys, or with locks and wheels which do not work properly, insufficient numbers of workers available to transfer patients from gurneys to the x-ray table, and having to handle weights above the shoulder level while handling equipment or positioning patients. All these factors might result in overload to the joints and spine^7^.

In a study with 37 orthopedic nursing professionals by Almeida and Lima^13^, 81% of the sample reported frequent musculoskeletal pain, particularly involving the lower back, legs, shoulders and neck, and 70% recurrent pain during the performance of daily tasks. In the study by Rocha et al.^14^ with 60 nursing technicians, the highest incidence of pain corresponded to the lower back, followed by the ankles, feet and shoulders. In their study with 76 health care workers Silva et al.^15^ found that pain most frequently involved the lower back, neck and shoulders. All these studies point to a high incidence of musculoskeletal problems, as also do our results.

In the present study, mental health problems were associated with large numbers of patients, poor working conditions, work involving sick individuals, poor cooperation among staff members, pressure for productivity, and stress derived from having a second job. A too large number of patients, conflict in interpersonal relationships, accelerated pace of work, shift work, having more than one job, physical and mental tiredness, discussions/intrigues and problems of communication have been mentioned as factors associated with mental health problems among health care workers^16^^,^^17^, as also the present study evidences.

A study described psychosocial factors of work which might cause emotional damage to radiology technicians, including burnout and lack of recognition, which are associated with distress among workers. Health care workers are subjected to considerable psychological pressure as a function of the very nature of their job, as e.g. having to cope with disease and death. Having to deal with diagnosis and suffering is a source of psychological conflict for radiology technicians^18^.

As the results show, most participants worked overtime and had a second job. These conditions, together with shift work, are liable to cause exhaustion and more particularly stress as a function of long working hours and difficulties to reconcile work with family and social life^19^^,^^20^.

Areosa suggests that having to deal with difficult situations, as e.g. providing care to multiple trauma victims and/or patients at high risk of death, are factors inherent to the work process of radiology technicians likely to cause exhaustion^21^.

Work-related respiratory disorders were mentioned as the third leading source of exhaustion, manifested as pneumonia, colds and rhinitis. In a study conducted at a hospital in Southern Brazil, in which the authors analyzed 1,050 reports relative to workers included in the nursing staff health monitoring system, respiratory diseases were the most prevalent, corresponding to 206 reports, and the second leading cause of sick leave^22^. In another study^23^, 12.5% of interviewed radiology technicians reported having suffered severe pneumonia. Respiratory diseases pose a continuous burden to health care workers and are one of the most frequent causes of illness among this population of workers. Laurell and Noriega observe that work burdens may interact and cause exhaustion^3^. On these grounds, one may draw a parallel between occurrence of stress and biological burdens, which might result in exhaustion associated with respiratory complaints.

Health care workers are continuously exposed to biological and mental burdens and risks. Radiology technicians are susceptible to contagion with infectious diseases, in addition to having to cope with situations characterized by high emotional tension, such as e.g. having to deal with people at high risk of death and multiple trauma victims, in addition to work overload^6^. Stress — described as mental exhaustion by the participants — interferes with the cortisol release into the bloodstream, with consequent changes in the levels of neutrophils and reduction of the number of lymphocytes, which together impair the body defenses^24^. This interaction between mental and biological burdens causes exhaustion, which manifests as a drop in immunity and thus might contribute to the occurrence of respiratory disorders, from colds to pneumonia.

Exhaustion often compels individuals to go to work even when sick, which corresponds to the phenomenon known as presenteeism^2^. Musculoskeletal, mental and respiratory disorders are described in the literature as health problems which might contribute to presenteeism^19^. To this, we should add the fact that work in health care is a source of risk by itself, and thus liable to cause exhaustion among workers. Presenteeism might lead to losses in productivity, job dissatisfaction, difficulties to perform movements required in job tasks, and impaired work ability^19^. Exhaustion may also make workers miss working days, which might interfere with the quality of care provision and thus result in financial losses^25^.

As concerns public policies in occupational health, we identified health problems among radiology technicians. Their participation in the present study contributed to improve the knowledge about sources of exhaustion. Our findings might help orient interventions on the work process and environment to promote a healthier workplace.

The limitations of the present study derive from the small number of participants, which hinders any attempt at generalization. More studies on how exhaustion develops among conventional radiology technicians are necessary.

## CONCLUSION

Based on the participants’ narratives we were able to identify the main sources of exhaustion within the work process of radiology technicians at a hospital in Santa Catarina, Brazil. The main sources of exhaustion were related to musculoskeletal disorders, mental exhaustion derived from daily stress, and respiratory diseases associated with impaired immunity and close contact with infectious agents. In addition, mental and biological burdens interact on a daily and might also influence the occurrence of illness.

The problems described by the participants are common to all health care workers in general, which fact indicates that the work process in this sector is influenced by definite particular aspects. Within this context, self-care should be a priority to achieve a healthier workplace. At the macro-organizational and political level, workers need to continuously vindicate their rights and fight for the implementation of guidelines included in resolutions, laws and other pieces of legislation on occupational health.

The present study makes visible sources of exhaustion within the work process of an occupational group still incipiently considered in the literature. Thus the results might serve to ground and boost additional studies addressing radiology technicians.
